# Supplementary material for: Sustained bacterial N2O reduction at acidic pH
Source: Nat Commun. 2024 May 15;15:4092. doi: 10.1038/s41467-024-48236-x (PMC11096178; doi:10.1038/s41467-024-48236-x)
Supplement: Supplementary file 1 — Supplementary Information [file 41467_2024_48236_MOESM1_ESM.pdf]

Supplementary Information

Sustained bacterial N_2O reduction at acidic pH

Guang He, Gao Chen, Yongchao Xie, Cynthia Swift, Diana Ramirez, Gyuhyon Cha, Konstantinos T. Konstantinidis, Mark Radosevich, and Frank E. Löffler

Corresponding author: Frank E. Löffler
Email: frank.loeffler@utk.edu

This PDF file includes:

Supplementary Notes 1-4
Supplementary Figures 1 to 12
Supplementary Tables 1 to 6
Supplementary References

Supplementary Notes 1: Phenotypic characterization of the N_2O-reducing co-culture. Cultivation experiments were performed in 160 mL glass serum bottles containing 100 mL of defined basal salt medium. The co-culture consumed 239 ± 1.16 µmol pyruvate within 7 days (Phase I) in the presence or absence of N_2O, and acetate (136 ± 6.05 µmol), formate (58.5 ± 3.33 µmol) and CO_2 (201 ± 5.53 µmol) were produced (Supplementary Fig. 1 A). In cultures without N_2O, formate was stable but was readily consumed when cultures were supplemented with N_2O (Supplementary Fig. 1 A, C, E). Measurable acetate consumption did not occur in any of the vessels indicating that acetate is not an electron donor for N_2O reduction (Supplementary Fig. 1 A, C, E).
In vessels that received pyruvate, H_2, and N_2O, H_2 and N_2O were simultaneously consumed following the depletion of pyruvate (day 10, Supplementary Fig. 1 B). N_2O consumption (Phase II) continued until H_2 became limiting but resumed when additional H_2 was provided (day 33, Supplementary Fig. 1 B). In cultures lacking N_2O, H_2 was stable, but consumption commenced immediately following the addition of N_2O (Supplementary Fig. 1 D). In replicate vessels without H_2, N_2O was consumed after day 10, apparently coupled to the oxidation of formate, a product of pyruvate fermentation (Supplementary Fig. 1 E, F). Pyruvate consumption (Phase I) and the production of formate, acetate and CO_2 as fermentation products preceded measurable consumption of N_2O and H_2 (or formate) (Phase II), suggesting that pyruvate was not a direct electron donor for N_2O reduction (Supplementary Fig. 1 A-F). Following an 18-day incubation period, triplicate cultures that received pyruvate (239 ± 1.16 µmol), H_2 (390 ± 1.66 µmol), and N_2O (378 ± 11.7 µmol) had completely consumed N_2O and nearly half of the exogenously added H_2 (143 ± 8.96 µmol) (Supplementary Fig. 1 B). Reduction of one molecule N_2O requires two electrons derived from the oxidation of one molecule of H_2 (i.e., $H_2 + N_2O \rightarrow H_2O + N_2$). The amount of H_2 oxidized did not reach the expected 1:1 stoichiometry, indicating that products from pyruvate fermentation (e.g., formate) serve as electron donors for N_2O reduction. Consistently, in cultures that received a low amount of pyruvate (i.e., 50 µmol), H_2 oxidation (361.2 ± 5.68 µmol) closely matched the amount of N_2O reduced (377 ± 3.53 µmol) (Supplementary Fig. 1 G). Pyruvate, H_2, and N_2O consumption were not apparent in vessels without inoculum (Supplementary Fig. 1 H). No growth occurred in cultures where CO_2 replaced pyruvate, suggesting no autotrophic activity (Supplementary Fig. 1 I).

Supplementary Notes 2: Consecutive transfers with N_2O as electron acceptor yield an N_2O-reducing co-culture. Shotgun metagenome sequencing performed on the original El Verde soil recovered 2,718 short-read fragments (150 bp) representing 16S rRNA genes, which could be assigned to 187 bacterial genera[1]. 16S rRNA gene fragments representing uncultured microbes, or not assigned to known genera in the El Verde soil sequence pools, accounted for 33.1% and 27.3% of the total 16S rRNA gene fragments, respectively. The remaining 16S rRNA gene fragments were assigned to *Acidothermus* (3.1%), '*Ca.* Solibacter' (2.0%) and '*Ca.* Udaeobacter' (7.4%). Taxa with less than 2% representation in the sequence pools are grouped as 'Others' and

accounted for 27% of the 16S rRNA gene fragments. Following six consecutive transfers (3%, v/v) of the El Verde soil microcosm in defined basal salt medium amended with pyruvate, H_2, and N_2O, all 16S rRNA amplicon sequences could be assigned to *Serratia* (68.0%), *Desulfosporosinus* (24.3%), *Desufitobacterium* (7.5%), *Caproiciproducens* (0.19%), *Peptoclostridium* (<0.05%) and *Lachnoclostridium* (<0.05%). Fifteen sequential transfers in the same medium reduced the microbial diversity to two populations, a *Serratia* sp. and *Desulfosporosinus* sp. Sanger sequencing of 16S rRNA gene clone libraries generated with DNA collected during Phase II from a 15th generation transfer culture revealed 16S rRNA gene fragments matching those of the *Serratia* sp. and *Desulfosporosinus* sp., and no other sequences were found. Deep metagenome sequencing of this co-culture yielded 387 Gbp of sequence data with a 2,865-fold coverage of the *Serratia* sp. genome and a 15,103-fold coverage of the *Desulfosporosinus* sp. genome, and 99.48% of the short-read sequences could be mapped to the contigs representing these two genomes. The *Serratia* genomes constructed from raw reads derived from co-culture EV and the axenic *Serratia* culture (494 Gbp of sequence data) were nearly identical (ANI 99.9%), indicating the assembly approach recovered nearly complete genomes of co-culture EV. The assembly of 16S rRNA gene fragments generated eight partial and two complete 16S rRNA genes, which shared between 91.1 to 99.5% sequence identity to the respective *Serratia* sp. and *Desulfosporosinus* sp. 16S rRNA gene sequences determined by Sanger sequencing (Supplementary Fig. 3). Both genomes possess a single 16S rRNA gene, and the observed differences in 16S rRNA sequences were attributed to errors associated with short-read assembly and sequencing. Phase contrast microscopy performed with co-culture EV suspension samples collected during Phase I and Phase II revealed the presence of the reported *Serratia* sp. and the *Desulfosporosinus* sp. cell morphologies[2,3]. Taken together, these observations support that 15 consecutive transfers yielded a co-culture comprising two bacterial populations, a *Serratia* sp. and a *Desulfosporosinus* sp.

Supplementary Notes 3: Isolation efforts. Numerous attempts were made to isolate the N_2O-reducing *Desulfosporosinus* sp. from co-culture EV. Serial 10-fold dilution-to-extinction series in pH 4.5 basal salt liquid medium amended with 2.5 mM pyruvate, 4.16 mM (nominal) N_2O, and 4.16 mM (nominal) H_2 recovered N_2O reduction activity from 10^{-6} dilution vials; however, the *Serratia* sp. was also present. The omission of pyruvate prevented growth of the *Serratia* sp. but without pyruvate, N_2O consumption did not commence. Plating of serially diluted culture suspension aliquots on solid TSA medium yielded uniform colonies. Sanger sequencing of PCR-amplified 16S rRNA genes of four single colonies from a 10^{-6} dilution plate yielded identical sequences with 100% sequence identity to the *Serratia* sp. Microscopic analysis revealed motile, short rods about 2 µm long representing *Serratia* cells (Supplementary Fig. 10 A) that grew readily in tryptic soy broth (TSB) medium. Growth also occurred in defined basal salt pH 4.5 medium amended with pyruvate, but the *Serratia* sp. did not consume H_2 or N_2O. Following

complete N_2O consumption in co-culture EV grown with 2.4 ± 0.01 mM pyruvate, 4.06 ± 1.16 mM (nominal) of H_2, and 3.96 ± 1.08 mM (nominal) of N_2O, *Desulfosporosinus* cells outnumbered *Serratia* cells approximately 5-fold. Microscopic observations also documented this population shift and *Desulfosporosinus* cells (rods about 6 µm in length) dominated the cell suspensions following N_2O consumption (Supplementary Fig. 10 B). Attempts to obtain isolated colonies of the *Desulfosporosinus* sp. in soft agar (0.8% low melting agarose, w/v) shake tubes were not productive because the reduction of N_2O generated N_2 gas bubbles preventing the recovery of isolated colonies (Supplementary Fig. 12). No growth was observed in medium bottles that received pasteurized inocula. Despite extensive efforts, the *Desulfosporosinus* sp. could not be separated from the *Serratia* sp. (also see Materials and Methods section).

Supplementary Notes 4: Basic genomic features of the populations in co-culture EV. For sequencing the *Desulfosporosinus nitrosoreducens* genome, genomic DNA was isolated from co-culture EV following complete consumption of N_2O, and the genome constructed from a metagenome dataset comprising 387 Gbp of raw reads. Construction of the *Serratia* sp. strain MF genome used pure culture DNA and 494 Gbp of raw reads.
Based on CheckM[4] estimates, the completeness of the *Serratia* and *Desulfosporosinus* genomes reached 99.8% and 99.5%, respectively (Supplementary Table 6). *Serratia* sp. strain MF and *Desulfosporosinus* sp. strain PR have 5,118,938 bp and 5,591,411 bp genomes, G+C contents of 58.8% and 44%, harbor 4,690 and 5,312 protein-coding genes, and possess 81 and 40 tRNA, and 10 and eight rRNA gene sequences, respectively. Genome-wide calculation of the average amino acid identity (AAI) showed that *Serratia* sp. strain MF shares 99.7% AAI with *Serratia marcescens* strain UMH3, suggesting these isolates are closely related. *Desulfosporosinus* sp. strain PR showed highest genomic relatedness with *Desulfosporosinus acidiphilus* strain SJ4 (79.5% AAI) and *Desulfosporosinus acididurans* strain M1 (79.4% AAI). *Desulfosporosinus* species are known for anaerobic sulfate reduction capacity; however, *Desulfosporosinsus* sp. strain PR has an incomplete dissimilatory sulfate reduction pathway, and genes for assimilatory sulfate reduction were absent. Gene clusters encoding two different nitrogenase complexes (*anf* and *nif* gene clusters) are exclusive to the *Desulfosporosinsus* sp. strain PR genome (Supplementary Fig. 7), indicating the ability to fix N_2. Although nitrogen fixation capability has never been experimentally validated in members of the genus *Desulfosporosinus*, their genetic makeup (i.e., complete *nif* gene clusters) suggest this to be a shared capability. Also present on the *Desulfosporosinsus* sp. strain PR genome is a *nor* gene cluster encoding nitric oxide (NO) reductase. Genes encoding nitrate and nitrite reductase are absent, consistent with the inability of *Desulfosporosinsus* sp. strain PR to utilize nitrate or nitrite as electron acceptors, although nitrate reduction was reported in some *Desulfosporosinsus* isolates[3,5,6].

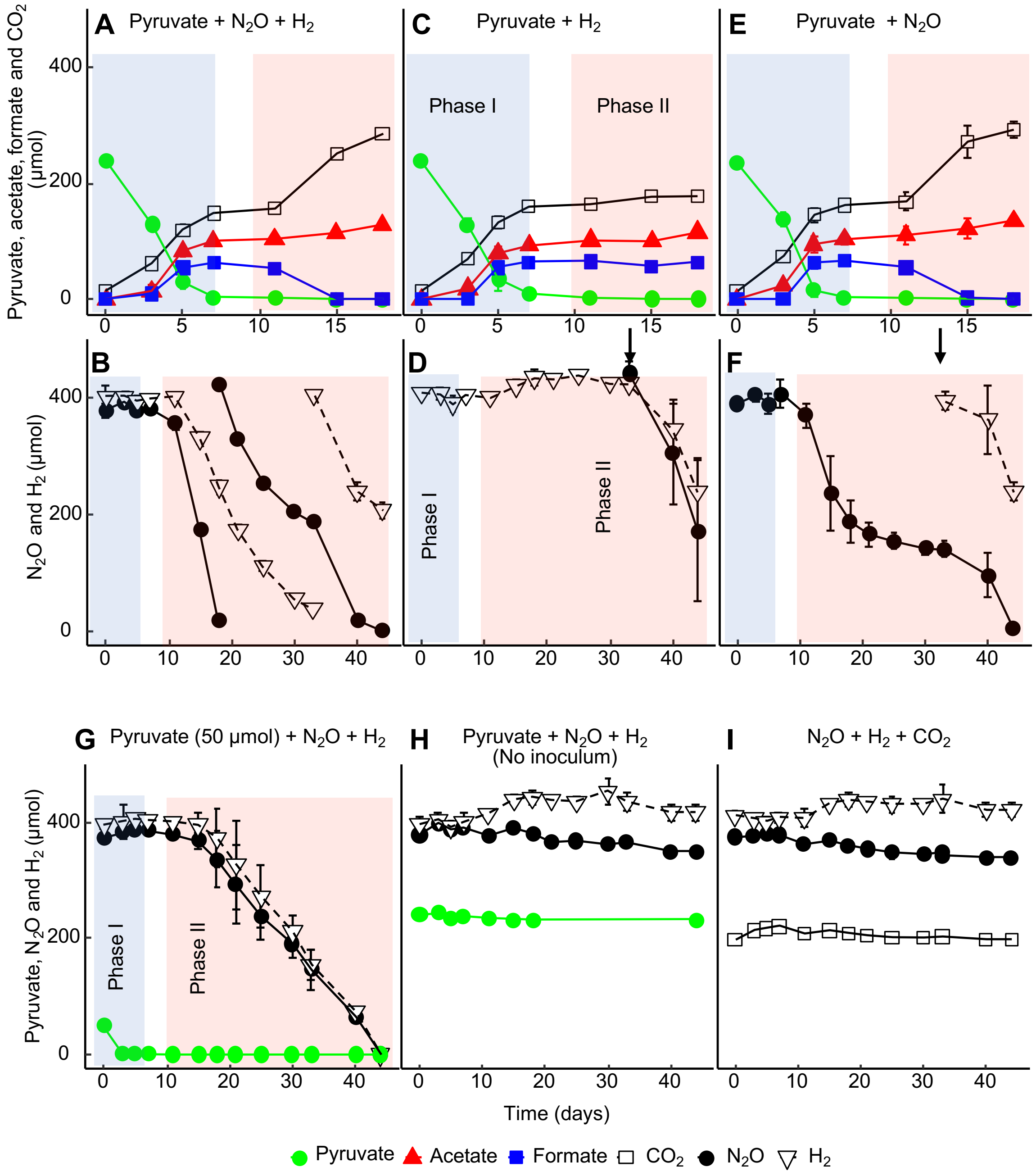

Supplementary Figure 1. Phenotypic characterization of co-culture EV. Cultures were grown in 160 mL glass serum bottles containing 100 mL of medium amended with pyruvate (50 or 250 µmol), H_2 (416 µmol) and N_2O (416 µmol) (A and B), pyruvate and H_2 (C and D), or pyruvate and N_2O (E and F). The arrow in panel D indicates the addition of N_2O to replicate vessels containing pyruvate and H_2. The arrow in panel F indicates the addition of H_2 to replicate vessels containing pyruvate and N_2O. Pyruvate consumption and associated product formation are documented in panels A, C, and E. N_2O and H_2 consumption are documented in panels B, D, F. Panel G shows

the performance of co-culture EV grown with a lower amount of pyruvate. Panel H depicts pyruvate, H_2 and N_2O amounts over time in vessels without inoculum. Autotrophic activity in cultures with CO_2, but lacking pyruvate, was not observed (panel I). The light blue shaded areas represent the pyruvate fermentation phase (Phase I), and the light red areas represent the N_2O reduction phase (Phase II). The data shown represent the averages of triplicate incubations and error bars represent the standard deviations (n=3). Error bars are not shown when smaller than the symbol.

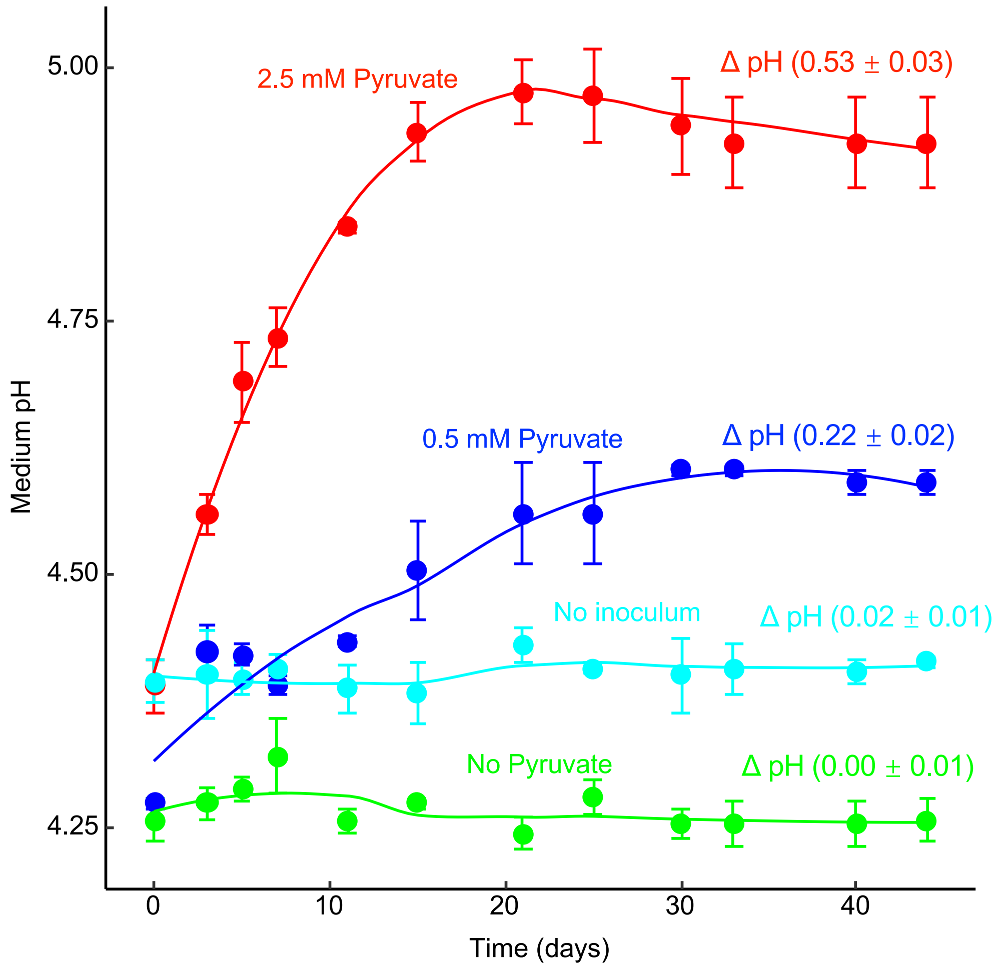

Supplementary Figure 2. Medium pH over the course of the incubation. pH profiles were measured in replicate co-culture EV incubation vessels that received 0, 0.5, or 2.5 mM pyruvate. pH changes (Δ pH) were calculated by subtracting the pH values measured immediately following inoculation from pH values measured at the end of incubation period. Pyruvate was not consumed in vessels that were not inoculated with co-culture EV (**Supplementary Fig. 1 H**). The lines are plotted based on fitting linear models using the ggplot2 package. The data shown are the averages of triplicate incubations and error bars represent the standard deviations (n=3). Error bars are not shown when smaller than the symbol.

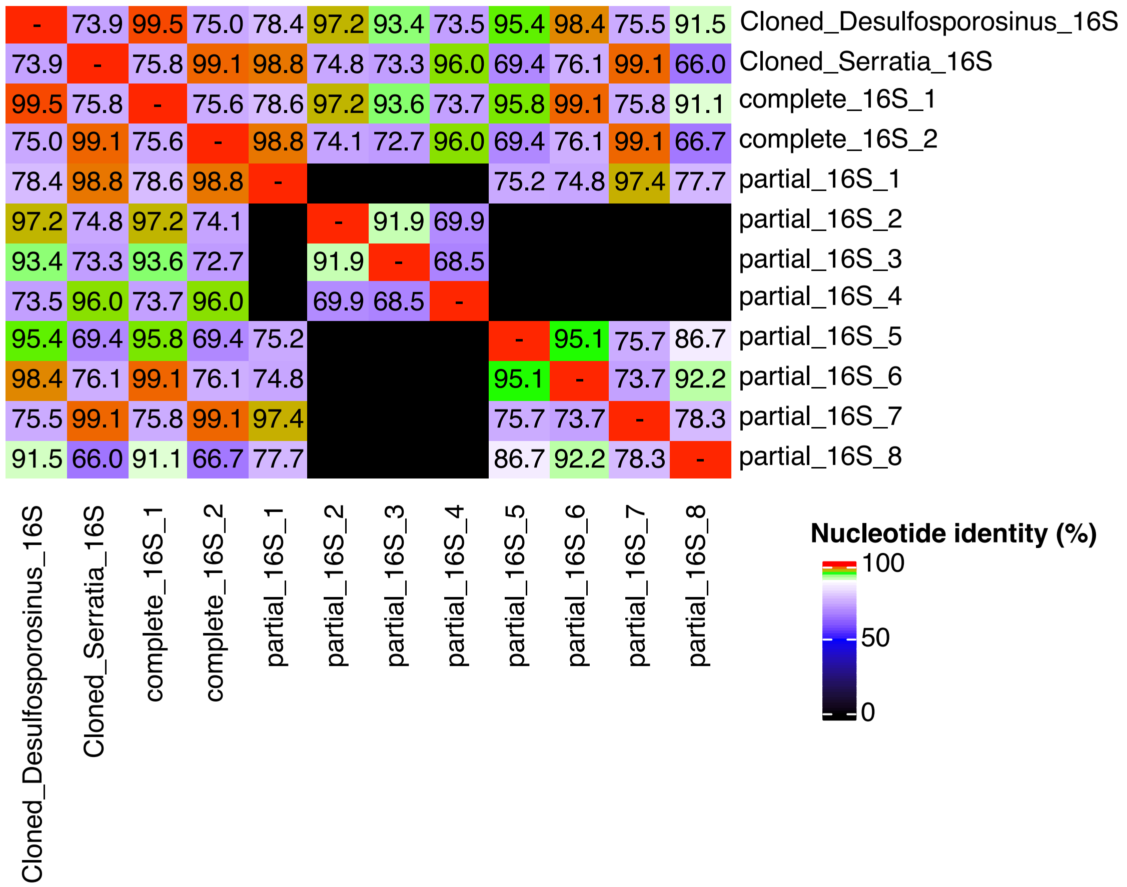

Supplementary Figure 3. Comparison of *Desulfosporosinus nitrosoreducens* strain PR and *Serratia* sp. strain MF 16S rRNA gene sequences derived from cloned 16S rRNA gene fragments (Sanger sequencing) and co-culture EV (metagenome sequencing). DNA extracted from co-culture EV following N_2O consumption served as template for the amplification of 16S rRNA gene fragments with general primer pair 8F and 1541R. The amplicons were cloned in *E. coli* and two uniform clone populations representing the 16S rRNA gene fragments of *Desulfosporosinus nitrosoreducens* strain PR (Cloned_Desulfosporosinus_16S) and of *Serratia* sp. strain MF (Cloned_Serratia_16S) were obtained. The assembly of metagenomic reads obtained from a 15th generation co-culture EV yielded full-length 16S rRNA genes of *Serratia* sp. strain MF (1,538 bp) and *Desulfosporosinus nitrosoreducens* strain PR (1,467 bp), and eight partial (< 500 bp)16S rRNA genes. Source data are provided as a Source Data file.

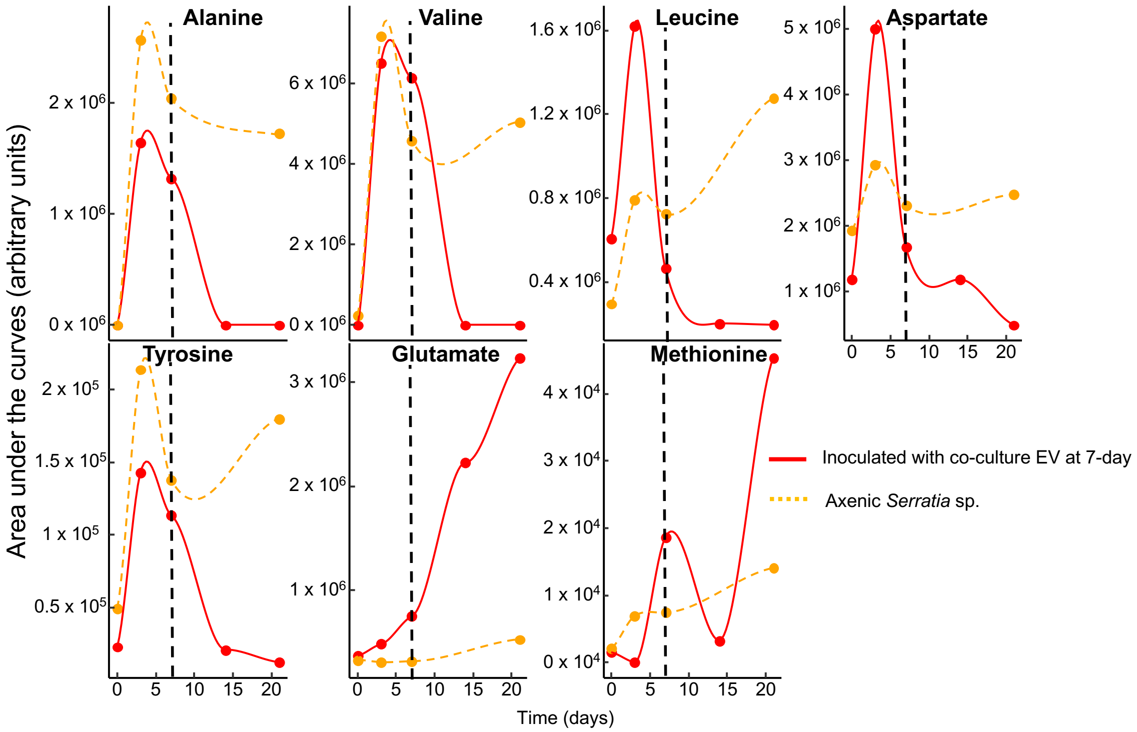

Supplementary Figure 4. Dynamic changes of amino acids in the supernatants of cultures following inoculation with *Serratia* sp. strain MF and with *Desulfosporosinus nitrosoreducens* strain PR (as co-culture EV). Axenic *Serratia* sp. strain MF cultures amended with pyruvate, N_2O, and H_2 received a co-culture EV inoculum, comprising *Serratia* sp. strain MF and *Desulfosporosinus nitrosoreducens* strain PR, on day 7 (indicated by the vertical dashed lines). Dashed orange lines represent amino acids in cultures inoculated with axenic *Serratia* sp. on day 0. Solid red lines represent amino acids in cultures inoculated with the axenic *Serratia* sp. on day 0 and subsequently inoculated with co-culture EV on day 7. The lines are plotted based on fitting linear models provided in the ggplot2 package. Note that axenic *Serratia* sp. cultures without co-culture EV inoculum cannot reduce N_2O. Shown are representative data obtained from a single culture in medium reduced with L-cysteine. Cystine was detected in cultures with L-cysteine added as reductant. In an independent experiment where DTT replaced L-cysteine as reductant, cystine was not detected, suggesting the *Serratia* sp. does not release cystine into the medium. Source data are provided as a Source Data file.

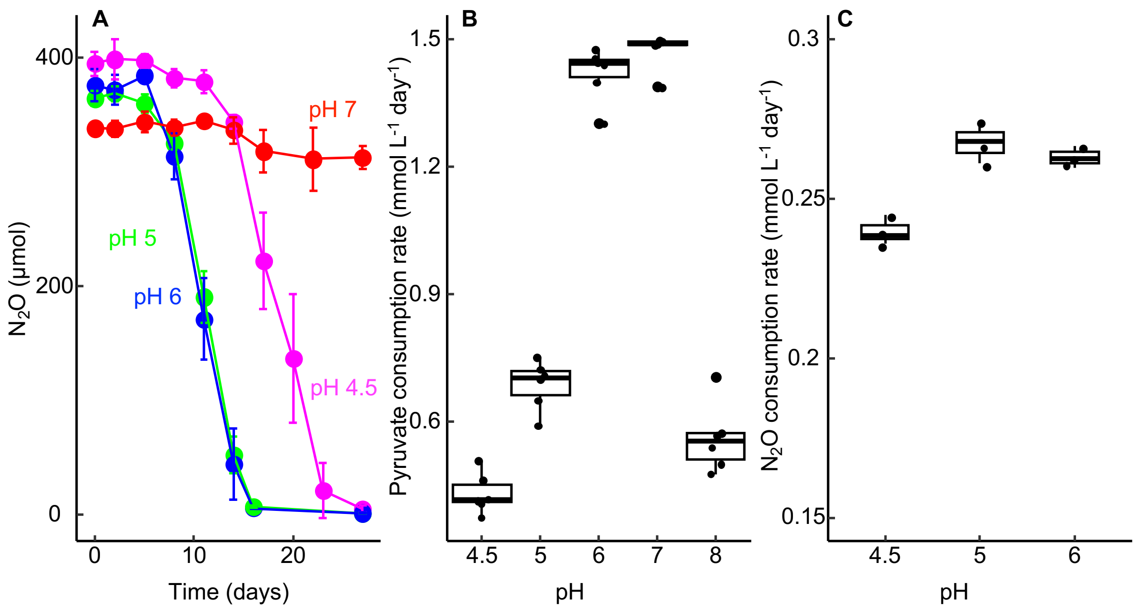

Supplementary Figure 5. Performance of co-culture EV at different medium pH values. (A) N_2O reduction was measured in triplicate 160 mL serum bottles containing 100 mL of medium (pH 3.5, 4.5, 5, 6, 7, 8) and inoculated with co-culture EV. N_2O reduction was observed between pH 4.5 and 6, but not at pH 3.5 and at or above pH 7. (B) Pyruvate consumption rates by *Serratia* sp. in cultures adjusted to pH 4.5, 5, 6, 7, and 8. Pyruvate was consumed in all incubation vessels except for those at pH 3.5. (C) N_2O consumption rates observed co-culture EV at pH 4.5, 5, and 6. No N_2O consumption was observed at pH 3.5, 7.0, and 8.0. Consumption rates of pyruvate and N_2O were calculated using data that fell within the linear ranges of the consumption curves. Each black dot in the box plots represent the consumption rates calculated from a single experiment. The data from replicate cultures were used for the calculations of pyruvate (n=6) and N_2O (n=3) consumption rates. Source data are provided as a Source Data file.

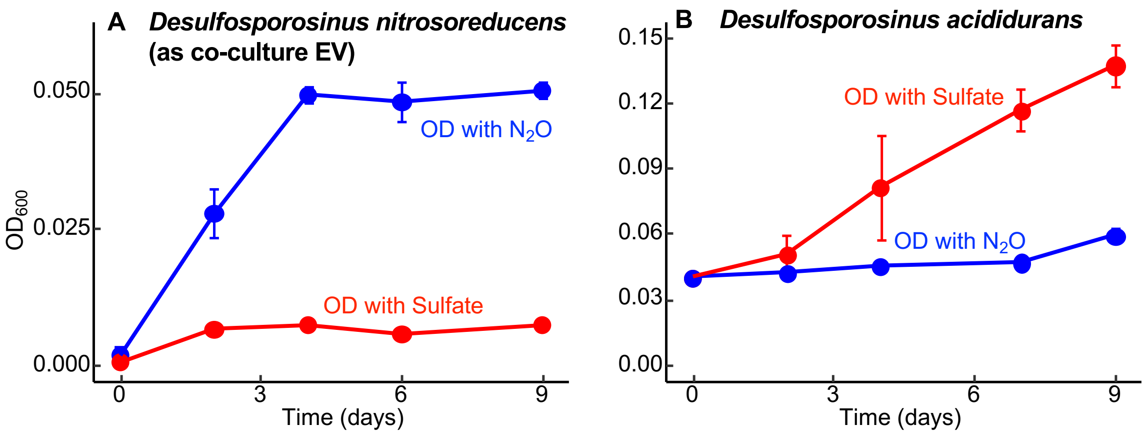

Supplementary Figure 6. Comparative growth studies with *Desulfosporosinus nitrosoreducens* strain PR (as co-culture EV) and *Desulfosporosinus acididurans* strain D. Panel A shows growth of *Desulfosporosinus nitrosoreducens* strain PR with 4.16 mM (nominal) N_2O (blue) or 5 mM SO_4^{2-} (red) as electron acceptor in pH 4.5 medium amended with the 15-amino acid mixture and 4.16 mM (nominal) H_2. Over the 9-day incubation period, strain PR completely consumed the initial dose of N_2O, whereas cultures amended with 5 mM SO_4^{2-} showed no growth or SO_4^{2-} consumption (4.97 ± 0.23 mM sulfate remained). OD values below 0.01 were measured in replicate cultures that did not receive N_2O as electron acceptor or H_2 as electron donor. Panel B compares growth of *Desulfosporosinus acididurans* strain D with 4.16 mM (nominal) N_2O (blue) or 5 mM SO_4^{2-} (red) as electron acceptors in pH 5.5 medium with 10 mM glycerol as electron donor. Over a 9-day incubation period, *Desulfosporosinus acididurans* strain D reduced 5 mM SO_4^{2-}, but N_2O was not consumed (4.10 ± 0.59 mM N_2O remained) in replicate cultures, even after an extended 20-day incubation period. Growth was monitored by measuring the optical density at 600 nm (OD_{600}). The data represent averages of triplicate incubations and error bars represent standard deviations (n=3). Error bars are not shown if smaller than the symbol. Source data are provided as a Source Data file.

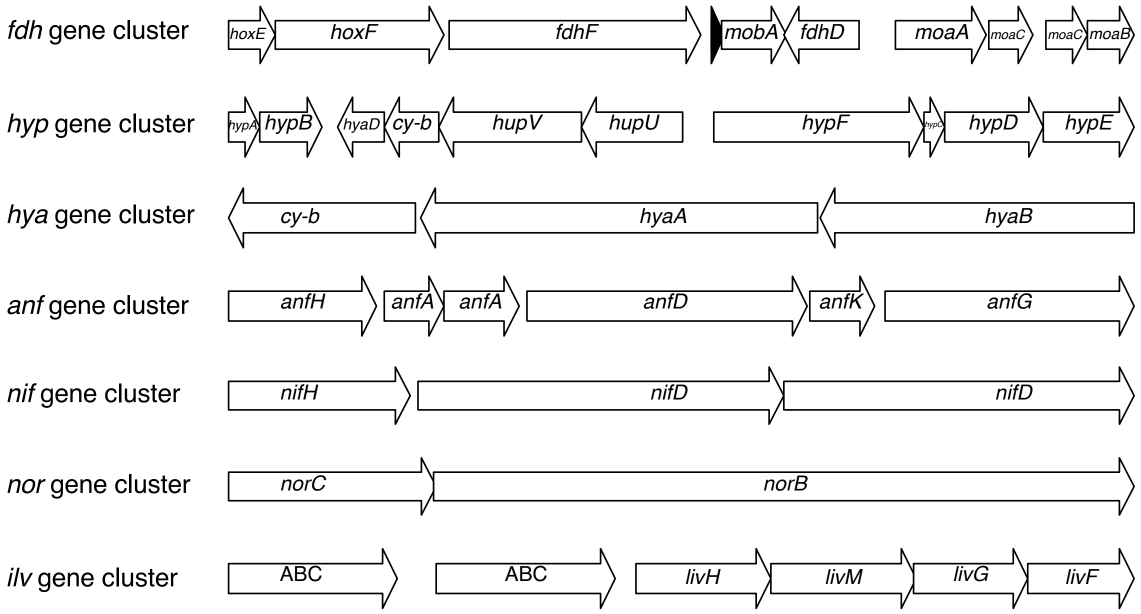

Supplementary Figure 7. Gene clusters related to N cycling found on the *Desulfosporosinus nitrosoreducens* strain PR genome (the *nos* gene cluster is shown Fig. 5). Displayed are formate dehydrogenase (*fdh* gene cluster), b-type Ni/Fe hydrogenase (*hyp* gene cluster), group 1 Ni/Fe hydrogenase (*hya* gene cluster), FeFe type nitrogenase (*anf* gene cluster), Mo-Fe nitrogenase (*nif* gene cluster), nitric-oxide reductase (*nor* gene cluster), high-affinity amino acid transport system (*ilv* gene cluster). The arrows indicate gene length and orientation. Preliminary annotation of coding genes was done with Prokka[7], MicrobeAnnotator[8], and the RAST server[9], and genes of interests were curated via BLAST analysis against the NCBI nr database. Cy-b: b-type cytochrome; ABC: ATP-binding cassette. The black arrow in the *fdh* gene cluster represents a gene encoding a small protein of unknown function. Source data are provided as a Source Data file.

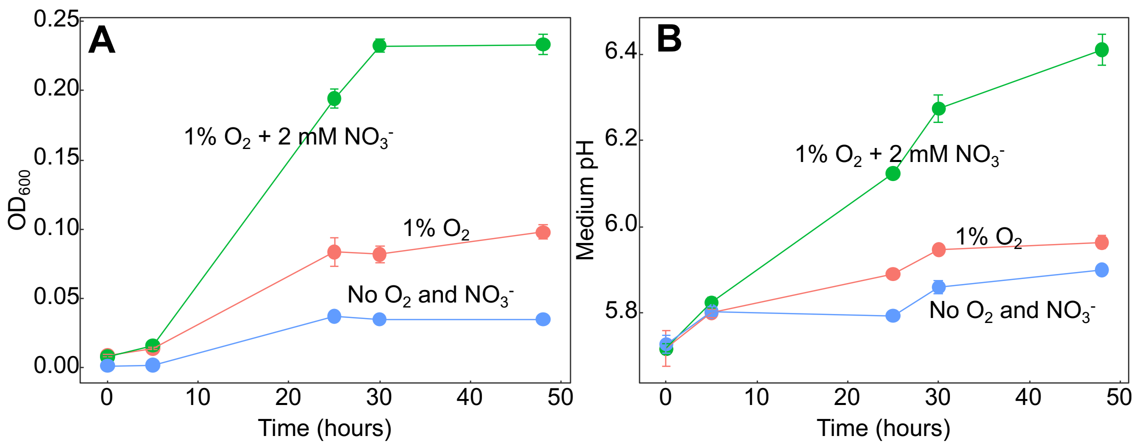

Supplementary Figure 8. pH changes during growth of a denitrifying mixed culture in 20 mM phosphate-buffered, 1/10-strength TSB medium supplemented with 2 mM $NaNO_3$. The 160 mL glass serum bottles containing 50 mL of medium received 5.2 mL of air to adjust the initial headspace oxygen to 1% (v/v). Panel A depicts growth of the denitrifying mixed culture, and Panel B shows the pH changes over a 48-hour incubation period. The initial medium pH was adjusted to 5.7 with 1.4 mM sulfuric acid, and all experimental conditions followed the procedure described for *Rhodanobacter* sp. strain C01[10]. Samples (1 mL) were periodically withdrawn to monitor OD_{600} and to measure pH with a pH electrode following removal of cells by centrifugation. *Rhodanobacter* sp. strain C01 is not available from type culture collections, and the experiment was performed with a denitrifying mixed culture obtained from acidic soil. The data represent averages of triplicate incubations and error bars represent standard deviations (n=3). Error bars are not shown if smaller than the symbol. Independent experiments corroborated these results. Source data are provided as a Source Data file.

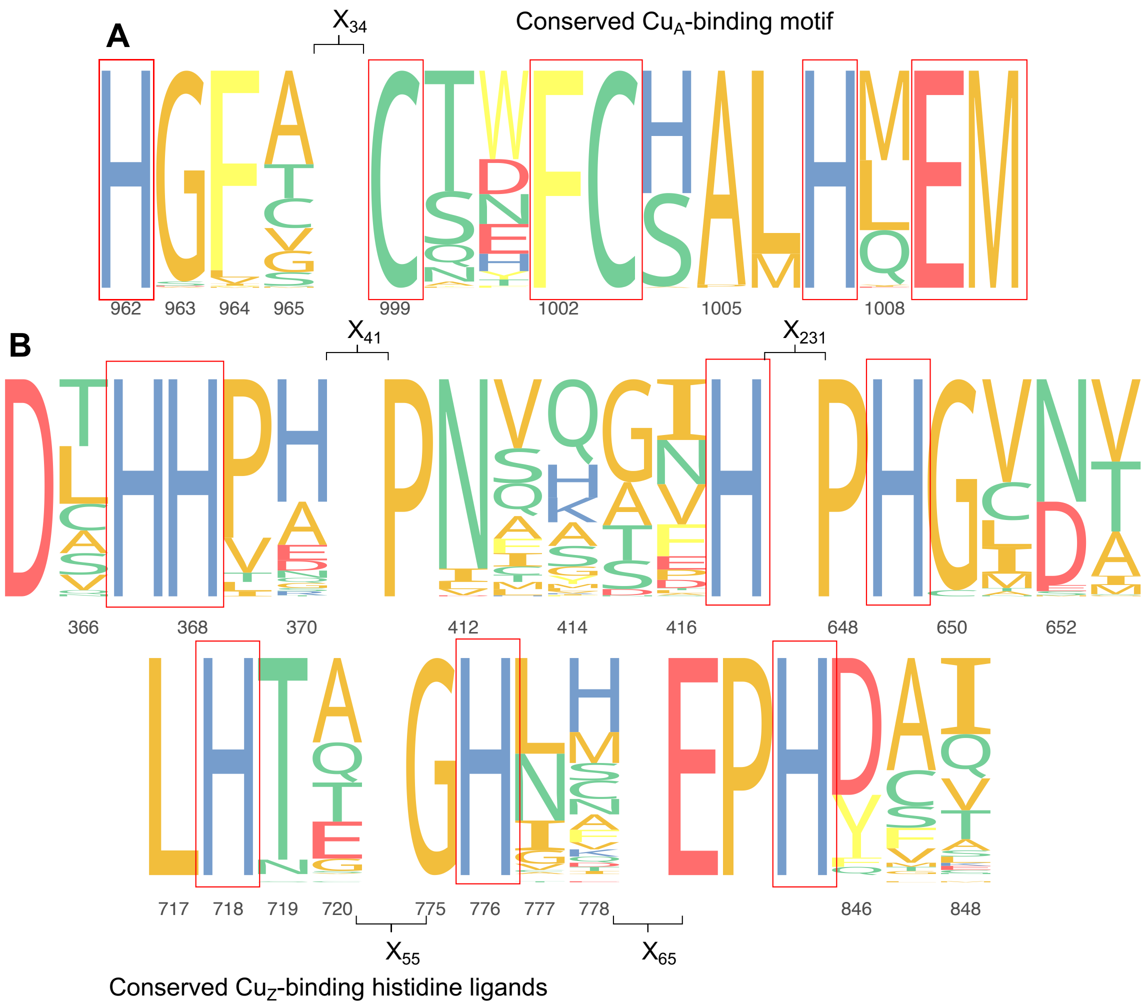

Supplementary Figure 9. Conserved features of NosZ of *Desulfosporosinus nitrosoreducens* strain PR and canonical clade I and clade II NosZ sequences. NosZ sequences were retrieved from a curated database (http://enve-omics.ce.gatech.edu/rocker/rocker/models/NosZ/NosZ.ref.fasta) and aligned using MAFFT[11]. (A) Conserved residues of the Cu_A copper-binding site involved in electron transfer. (B) Seven conserved histidine residues involved in copper binding at the catalytic Cu_Z site lack a conserved sequence motif. The X_n values indicate the number of residues between conserved residues at the Cu_A and Cu_Z sites, and the red boxes indicate residues involved in copper binding based on protein crystallography data[12]. The height of symbols (i.e., the size of letter abbreviations for amino acids) reflects the relative frequency of the amino acid at that position. The sequence logo was created with ggmsa v1.0.3[13]. Source data are provided as a Source Data file.

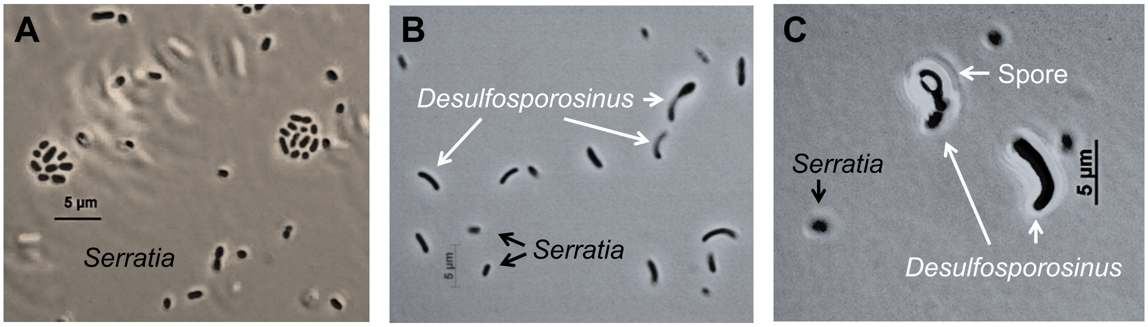

Supplementary Figure 10. Light microscopic images showing the morphologies of *Serratia* sp. strain MF and *Desulfosporosinus nitrosoreducens* strain PR cells. (A) Axenic culture of *Serratia* sp. strain MF. (B) Co-culture EV comprising *Serratia* sp. strain MF and *Desulfosporosinus nitrosoreducens* strain PR. (C) Four-week-old co-culture EV showing spore formation in *Desulfosporosinus nitrosoreducens* strain PR cells.

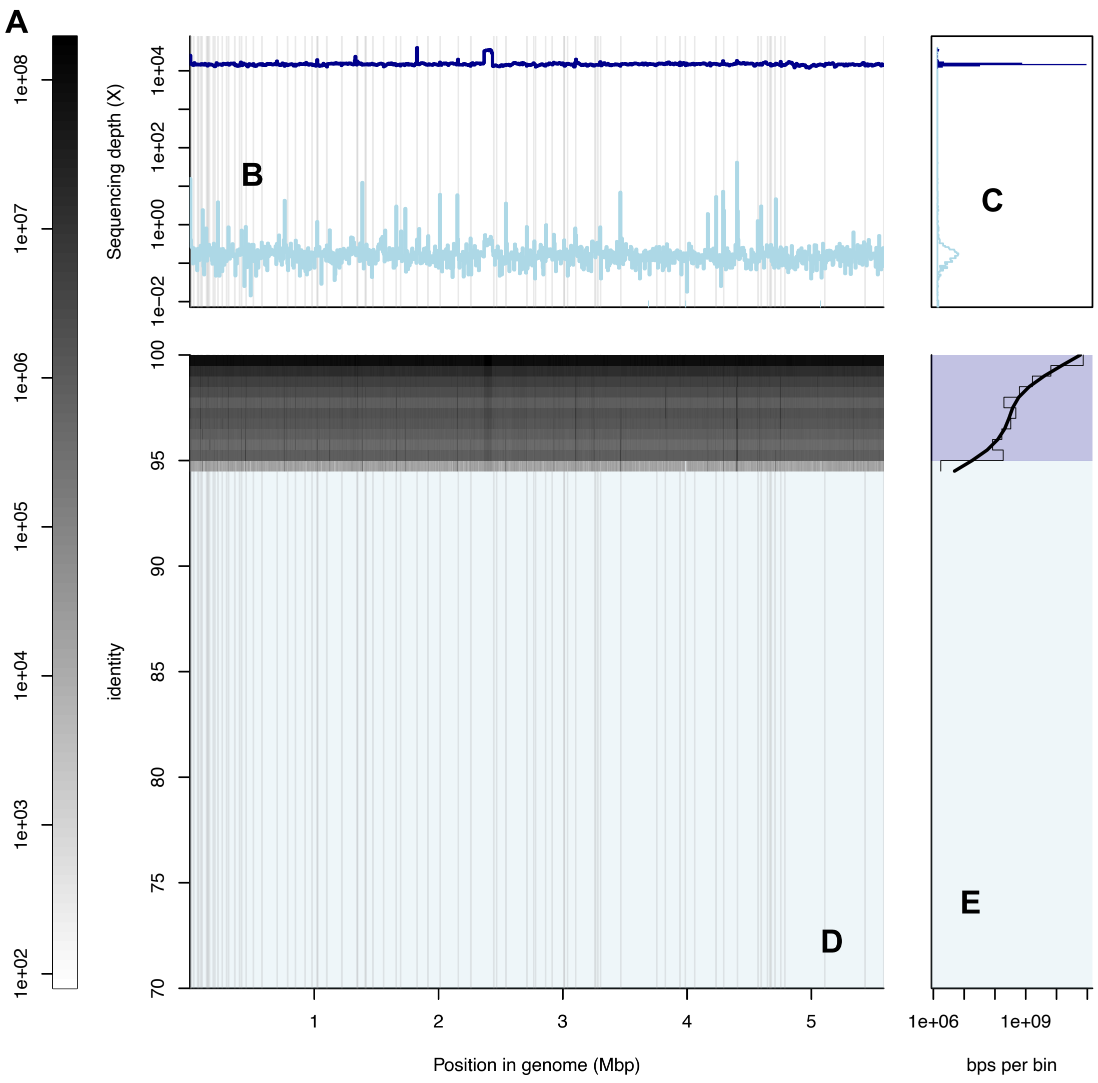

Supplementary Figure 11. Fragment recruitment plot of the *Desulfosporosinus nitrosoreducens* strain PR genome to the metagenome dataset derived from the 15th generation transfer co-culture EV. The recruitment was performed via processing a BLAST search of the metagenome fragments against the *Desulfosporosinus nitrosoreducens* strain PR genome. The tabular BLAST result was parsed using BLASTab.catsbj.pl, and graphical representation was generated with the BlasTab.recplot2.R embedded in the Enveomics collection[14]. (A) The bar on the left shows the number of fragments with different identity recruited to each position on the genome. (B) The sequencing depth across the *Desulfosporosinus nitrosoreducens* strain PR genome is shown on a logarithmic scale. (C) Sequencing depth histogram with peaks from values above 95% identity automatically identified as skewed normal distribution. (D) Metagenome fragments recruited to the *Desulfosporosinus nitrosoreducens* strain PR genome, placed by location (x-axis) and identity (y-axis). (E) Identity histogram of mapping fragments (light gray) and smoothed spline (black).

The backgrounds in panels D and E, and the line colors in panels B and C, correspond to the identity matches above (dark blue) and below (light blue) 95 %. A recruitment plot of the *Desulfosporosinus nitrosoreducens* strain PR genome to the metagenome datasets derived from El Verde soil is not shown as the covered fraction of the 5.6 Mbp genome was below 10% (i.e., 0.56 Mbp).

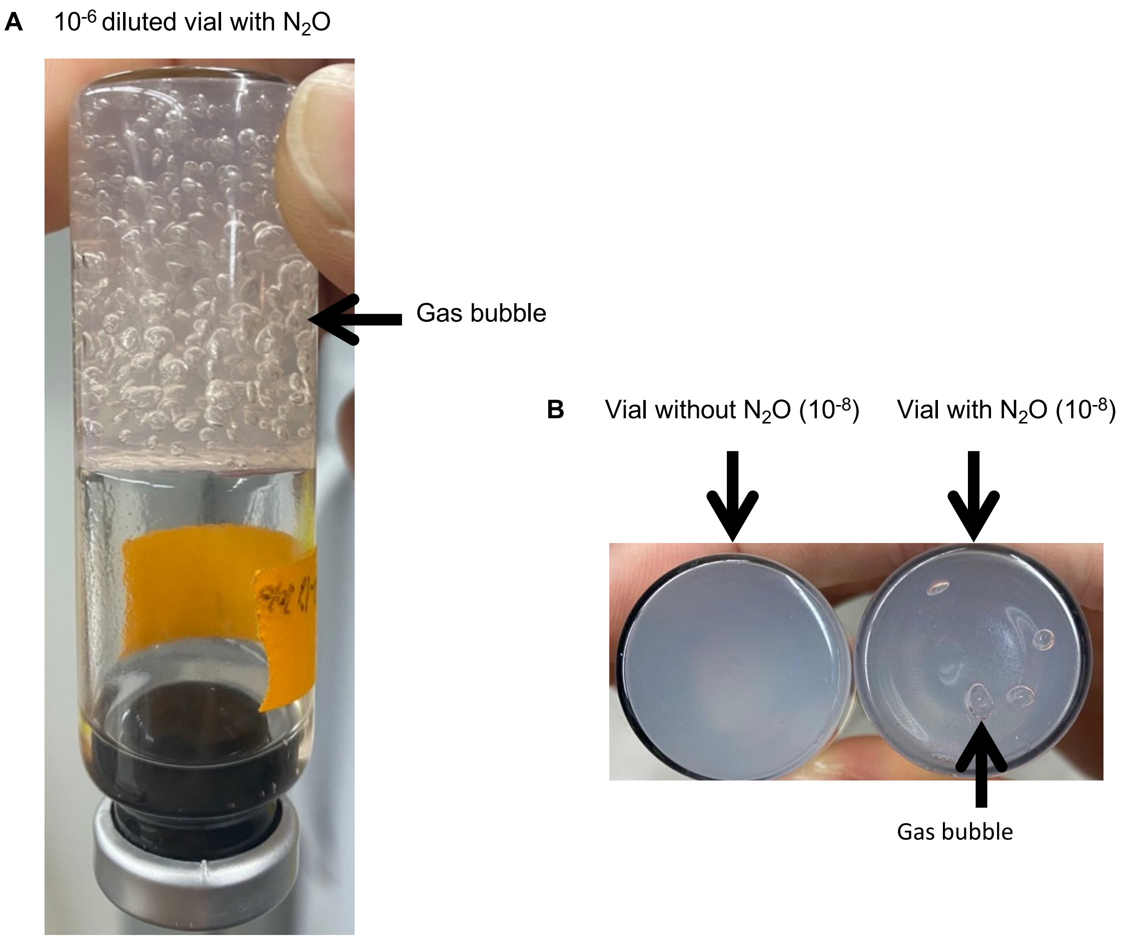

Supplementary Figure 12. Formation of gas bubbles in soft agar shake tubes. (A) Visible gas bubbles (presumably N_2) following N_2O consumption. The vials were incubated with the stoppers down for 28 days. (B) The formation of gas bubbles was strictly dependent on the presence of N_2O, and no bubbles formed in replicate vials without N_2O. No gas bubbles formed in control incubations without inoculum.

Supplementary Table 1. Amino acids and concentrations used to augment the growth medium for cultivation of *Desulfosporosinus nitrosoreducens*. [a]

Amino acid	Amino acid concentration (μM)		Carbon (μmol 100 mL medium^{-1})
Alanine	56		16.8
Valine	77		38.5
Aspartate	82		33.1
Isoleucine	38		22.9
Methionine	27		13.4
Tyrosine	16		14.9
Histidine	19		11.6
Tryptophan	10		10.8
Glutamate	68		34.0
Proline	86		43.5
Arginine	28		17.2
Glycine	40		8.0
Threonine	42		16.8
Lysine	69		41.1
Serine	76		22.9
		Total carbon	383.7

[a] The addition of individual amino acids did not stimulate N_2O-dependent growth of *Desulfosporosinus nitrosoreducens*. Similarly, a 5-amino acid mixture comprising alanine, valine, leucine, aspartate, and tyrosine did not promote N_2O consumption. Weak and incomplete N_2O reduction was observed in cultures that received a 6-amino acid mixture comprising alanine, valine, leucine, aspartate, tyrosine, and methionine. The 15-amino acid mixture (i.e., all amino acids listed in Supplementary Table 1) supported growth of *Desulfosporosinus nitrosoreducens* via hydrogenotrophic N_2O reduction.

Supplementary Table 2. qPCR assays targeting the 16S rRNA genes of *Serratia* sp. strain MF and *Desulfosporosinus nitrosoreducens* strain PR.

Target	Primer sequence	Probe
Serratia sp. strain MF	TGCTACAATTGGCGTATACAA (Ser_1316qF)<br>GACTACGACATACTTTATGA (Ser_1386qR)	None
Desulfosporosinus nitrosoreducens strain PR	TGCTACAATGGCCGGTACAG (DS_1316qF)<br>CGAACTGAGACCGGCTTTCT (DS_1388qR)	FAM-AAGCTGTGAAGTGGAGCCAATC-MGB [a]

[a] FAM: 6-Carboxyfluorescein attached to the 5’ end of the probe. MGB: Minor Groove Binder attached to the 3’ end of the probe.

Supplementary Table 3. Sequence information submitted to the NCBI database.

Dataset	Data type	Accession No.
Enrichment culture (6th transfer)	Amplicon (16S V3-V4)	SRR24215177
Enrichment culture (9th transfer)	Amplicon (16S V3-V4)	SRR24083098
Co-culture EV (15th transfer)	SRA [a]	SRR24709127
Serratia sp. (isolate)	SRA [a]	SRR24709126
16S rRNA gene (*Desulfosporosinus*)	Complete gene	OR076434
16S rRNA gene (*Serratia*)	Complete gene	OR076433
Desulfosporosinus nitrosoreducens strain PR	GenBank Assembly [b]	GCA_030954495.1
Serratia sp. strain MF	GenBank Assembly [b]	GCA_030954505.1

[a] SRA: Sequence Read Archive (raw sequence data)

[b] Genome assembly constructed from SRA data

Supplementary Table 4. Genomes of select bacteria with representative *nos* clusters (see Figure 5 in the manuscript), NosZ type (clade), and reported pH range of N_2O reduction activity.

**Organism**	**Genome NCBI acc no.**	**Reported pH range for N_2O reduction**	***nosZ* clade**	**Reference**
Desulfosporosinus nitrosoreducens strain PR	GCA_030954495.1	4.5-6.0	II	This work
Desulfosporosinus meridiei strain DSM 13257	NC_018515.1	ND	II	[15]
Nitratiruptor labii strain HRV44	AP022826.1	5.4-6.4	II	[16]
Anaeromyxobacter dehalogenans strain 2CP-1	NC_011891.1	~7	II	[15]
Ferroglobus placidus	NC_013849.1	ND	II	[15]
Azospira oryzae	GCA_905120365.1	ND	II	[15]
Paracoccus denitrificans strain DSM 413	AL591688.1	7-8	I	[17]
Shewanella loihica strain PV-4	NC_009092.1	6-8	I	[18]

Supplementary Table 5. Genomes used for phylogenomic analyses and the classification of *Desulfosporosinus nitrosoreducens*.

Organism	NCBI accession No. of genome
Desulfosporosinus nitrosoreducens strain PR	GCA_030954495.1
Desulfosporosinus youngiae DSM 17734	NZ_CM001441.1
Desulfosporosinus orientis DSM 765	NC_016584.1
Desulfosporosinus metallidurans strain OL	NZ_MLBF01000001.1
Desulfosporosinus meridiei DSM 13257	NC_018515.1
Desulfosporosinus lacus DSM 15449	NZ_FQXJ01000052.1
Desulfosporosinus hippie DSM 8344	NZ_fncp01000067.1
Desulfosporosinus fructosivorans strain 63.6F	NZ_SPQQ01000010.1
Desulfosporosinus acidiphilus SJ4	NC_018068.1
Desulfosporosinus acididurans strain M1	NZ_LDZY01000001.1
'*Ca.* Desulfosporosinus infrequens'	NZ_OMOF01000971.1
Syntrophobotulus glycolicus DSM 8271	NC_015172.1
Desulfitobacterium metallireducens DSM 15288	NZ_CP007032.1
Desulfitobacterium hafniense DCB-2	NC_011830.1
Desulfitobacterium dichloroeliminans LMG P-21439	NC_019903.1
Desulfitobacterium dehalogenans ATCC 51507	NC_018017.1
Desulfitobacterium chlororespirans DSM 11544	NZ_FRDN01000033.1
Dehalobacter restrictus strain E1	CANE01000102.1
Dehalobacter restrictus strain 12DCA	NZ_CP046996.1
Dehalobacter restrictus DSM 9455	NZ_CP007033.1

Supplementary Table 6. Genome features of *Desulfosporosinus nitrosoreducens* strain PR and *Serratia* sp. strain MF, and comparison to closest relatives.

	**Organism**				
**Feature**	**(1)**	**(2)**	**(3)**	**(4)**	**(5)**
Genome size (bp)	5,591,411	4,991,181	4,637,866	5,118,938	5,300,955
Completeness (%) [a]	99.5	99.1	92.3	98.0	95.6
Contamination (%) [a]	5.7	0.0	5.14	0.53	4.03
GC content (%)	44	42	41.5	58.8	59.5
5S rRNA genes	8	8	8	8	8
16S rRNA genes	1	9	10 [b]	1	7
23S rRNA genes	1	8	11 [b]	1	7
tRNA genes	105	66	67	81	91
Coding sequences	5,312	4,554	4,317	4,690	4,941
Accession number	GCA_030954495.1	GCA_000255115.3	GCA_001029285.1	GCA_030954505.1	GCF_002220655.1

(1) *Desulfosporosinus nitrosoreducens* strain PR (this study)

(2) *Desulfosporosinus acidiphilus* strain SJ4 [19]

(3) *Desulfosporosinus acididurans* strain M1 [3]

(4) *Serratia* sp. strain MF (this study)

(5) *Serratia marcescens* strain UMH3 [20]

[a] Genome completeness and contamination were evaluated based on the presence of 120 single copy genes using CheckM.

[b] Only partial 16S and 23S rRNA genes were found on the *Desulfosporosinus acididurans* strain M1 genome.

Supplementary References

1 Karthikeyan, S. *et al.* Metagenomic characterization of soil microbial communities in the Luquillo experimental forest (Puerto Rico) and implications for nitrogen cycling. *Appl. Environ. Microbiol.*, AEM.00546-00521 (2021). https://doi.org:10.1128/aem.00546-21

2 Keawmanee, P., Rattanakreetakul, C. & Pongpisutta, R. Microbial reduction of fumonisin B1 by the new isolate *Serratia marcescens* 329-2. *Toxins* **13**, 638 (2021).

3 Sánchez-Andrea, I., Stams, A. J. M., Hedrich, S., Ňancucheo, I. & Johnson, D. B. *Desulfosporosinus acididurans* sp. nov.: an acidophilic sulfate-reducing bacterium isolated from acidic sediments. *Extremophiles* **19**, 39-47 (2015). https://doi.org:10.1007/s00792-014-0701-6

4 Parks, D. H., Imelfort, M., Skennerton, C. T., Hugenholtz, P. & Tyson, G. W. CheckM: assessing the quality of microbial genomes recovered from isolates, single cells, and metagenomes. *Genome Res.* **25**, 1043-1055 (2015).

5 Vatsurina, A., Badrutdinova, D., Schumann, P., Spring, S. & Vainshtein, M. *Desulfosporosinus hippei* sp. nov., a mesophilic sulfate-reducing bacterium isolated from permafrost. *Int. J. Syst. Evol. Microbiol.* **58**, 1228-1232 (2008). https://doi.org:https://doi.org/10.1099/ijs.0.65368-0

6 Hippe, H. & Stackebrandt, E. in *Bergey's Manual of Systematics of Archaea and Bacteria* 1-10 (2015).

7 Seemann, T. Prokka: rapid prokaryotic genome annotation. *Bioinformatics* **30**, 2068-2069 (2014). https://doi.org:10.1093/bioinformatics/btu153

8 Ruiz-Perez, C. A., Conrad, R. E. & Konstantinidis, K. T. MicrobeAnnotator: a user-friendly, comprehensive functional annotation pipeline for microbial genomes. *BMC Bioinformatics* **22**, 11 (2021). https://doi.org:10.1186/s12859-020-03940-5

9 Overbeek, R. *et al.* The SEED and the Rapid Annotation of microbial genomes using Subsystems Technology (RAST). *Nucleic Acids Res.* **42**, D206-D214 (2013). https://doi.org:10.1093/nar/gkt1226

10 Lycus, P. *et al.* Phenotypic and genotypic richness of denitrifiers revealed by a novel isolation strategy. *ISME J.* **11**, 2219-2232 (2017). https://doi.org:10.1038/ismej.2017.82

11 Katoh, K. & Standley, D. M. MAFFT multiple sequence alignment software version 7: improvements in performance and usability. *Mol. Biol. Evol.* **30**, 772-780 (2013). https://doi.org:10.1093/molbev/mst010

12 Zhang, L., Wüst, A., Prasser, B., Müller, C. & Einsle, O. Functional assembly of nitrous oxide reductase provides insights into copper site maturation. *Proc. Natl. Acad. Sci. U.S.A.* **116**, 12822-12827 (2019). https://doi.org:10.1073/pnas.1903819116

13 Zhou, L. *et al.* ggmsa: a visual exploration tool for multiple sequence alignment and associated data. *Brief. Bioinform.* **23** (2022). https://doi.org:10.1093/bib/bbac222

14 Rodriguez-R, L. M. & Konstantinidis, K. T. The enveomics collection: a toolbox for specialized analyses of microbial genomes and metagenomes. *PeerJ Preprints* (2016).

15 Sanford, R. A. *et al.* Unexpected nondenitrifier nitrous oxide reductase gene diversity and abundance in soils. *Proc. Natl. Acad. Sci. U.S.A.* **109**, 19709-19714 (2012). https://doi.org:10.1073/pnas.1211238109

16 Fukushi, M. *et al.* Biogeochemical implications of N_2O-reducing thermophilic *Campylobacteria* in deep-sea vent fields, and the description of *Nitratiruptor labii* sp. nov. *iScience* **23**, 101462 (2020). https://doi.org:https://doi.org/10.1016/j.isci.2020.101462

17 Bergaust, L., Mao, Y., Bakken Lars, R. & Frostegård, Å. Denitrification response patterns during the transition to anoxic respiration and posttranscriptional effects of suboptimal pH on nitrogen oxide reductase in *Paracoccus denitrificans*. *Appl. Environ. Microbiol.* **76**, 6387-6396 (2010). https://doi.org:10.1128/AEM.00608-10

18 Kim, H., Park, D. & Yoon, S. pH control enables simultaneous enhancement of nitrogen retention and N_2O reduction in *Shewanella loihica* Strain PV-4. *Front. Microbiol.* **8** (2017). https://doi.org:10.3389/fmicb.2017.01820

19 Alazard, D., Joseph, M., Battaglia-Brunet, F., Cayol, J.-L. & Ollivier, B. *Desulfosporosinus acidiphilus* sp. nov.: a moderately acidophilic sulfate-reducing bacterium isolated from acid mining drainage sediments. *Extremophiles* **14**, 305-312 (2010). https://doi.org:10.1007/s00792-010-0309-4

20 Dabos, L. *et al.* SME-4-producing *Serratia marcescens* from Argentina belonging to clade 2 of the *S. marcescens* phylogeny. *J. Antimicrob. Chemother.* **74**, 1836-1841 (2019). https://doi.org:10.1093/jac/dkz115
