# Supplementary material for: Sustained bacterial N2O reduction at acidic pH
Source: Nat Commun. 2024 May 15;15:4092. doi: 10.1038/s41467-024-48236-x (PMC11096178; doi:10.1038/s41467-024-48236-x)
Supplement: Supplementary file 3 — Description of Additional Supplementary Files [file 41467_2024_48236_MOESM3_ESM.pdf]

## Description of Additional Supplementary Files:

**Supplementary Dataset 1:** Custom library containing 279 metabolite formulas used for metabolome analysis (Biological and Small Molecule Mass Spectrometry Core, University of Tennessee Knoxville).
