# Supplementary material for: Sustained bacterial N2O reduction at acidic pH
Source: Nat Commun. 2024 May 15;15:4092. doi: 10.1038/s41467-024-48236-x (PMC11096178; doi:10.1038/s41467-024-48236-x)
Supplement: Supplementary file 5 — Reporting Summary [file 41467_2024_48236_MOESM5_ESM.pdf]

nature portfolio

Corresponding author(s): Frank E. Löffler

Last updated by author(s): Apr 17, 2024

Reporting Summary

Nature Portfolio wishes to improve the reproducibility of the work that we publish. This form provides structure for consistency and transparency in reporting. For further information on Nature Portfolio policies, see our Editorial Policies and the Editorial Policy Checklist.

Statistics

For all statistical analyses, confirm that the following items are present in the figure legend, table legend, main text, or Methods section.

n/a	Confirmed	
☐	☒	The exact sample size (*n*) for each experimental group/condition, given as a discrete number and unit of measurement
☐	☒	A statement on whether measurements were taken from distinct samples or whether the same sample was measured repeatedly
☐	☒	The statistical test(s) used AND whether they are one- or two-sided<br>*Only common tests should be described solely by name; describe more complex techniques in the Methods section.*
☒	☐	A description of all covariates tested
☒	☐	A description of any assumptions or corrections, such as tests of normality and adjustment for multiple comparisons
☐	☒	A full description of the statistical parameters including central tendency (e.g. means) or other basic estimates (e.g. regression coefficient) AND variation (e.g. standard deviation) or associated estimates of uncertainty (e.g. confidence intervals)
☐	☒	For null hypothesis testing, the test statistic (e.g. *F*, *t*, *r*) with confidence intervals, effect sizes, degrees of freedom and *P* value noted<br>*Give P values as exact values whenever suitable.*
☒	☐	For Bayesian analysis, information on the choice of priors and Markov chain Monte Carlo settings
☒	☐	For hierarchical and complex designs, identification of the appropriate level for tests and full reporting of outcomes
☒	☐	Estimates of effect sizes (e.g. Cohen's *d*, Pearson's *r*), indicating how they were calculated

Our web collection on statistics for biologists contains articles on many of the points above.

Software and code

Policy information about availability of computer code

Data collection	Basic Local Alignment Search Tool (BLAST,https://blast.ncbi.nlm.nih.gov/Blast.cgi), Enveomics Collection Tools (https://blast.ncbi.nlm.nih.gov/Blast.cgi), ROCker (http://enve-omics.ce.gatech.edu/rocker/), nf-core/ampliseq v2.3.1, nf-core/MAG v2.1.0, Metabolomic Analysis and Visualization Engine (MAVEN), Agilent 1200 Series high-performance liquid chromatography (HPLC) system (Palo Alto, CA, USA), Agilent 3000A Micro-Gas Chromatograph (Palo Alto, CA, USA), Appplied Biosystems ViiA 7 system (Applied Biosystems), Illumina NovaSeq 6000 platform, Exactive Plus Orbitrap MS (Thermo Scientific),
Data analysis	R studio v2023.06.1+524, FastQC v0.11.9, fastp v0.20.1, Bowtie2 v2.4.2, Megahit2 v1.2.9, MetaBAT2 v2.15, CheckM v1.2.2, MetaGeneMark-2, MicrobeAnnotator v2.0.5, ncbi-BLAST v2.12.0, RAST server (https://rast.nmpdr.org), KofamKOALA(https://www.genome.jp/tools/kofamkoala/), Enveomics Collection tools, Nonpareil v3.4.1, cblaster v1.3.18, DeepTMHMM v1.0.24, GTDB-TK v1.3.0, EzAAI v1.2.2, ggtree v3.10.0, ggtreeExtra v3.18, MAFFT v7.407, RAxML-NG v1.2.1, ModelTest-NG v0.1.7, ggmsa v1.8.0, ggplot2 v3.4.4, ComplexHeatmap v2.18.0, gggenes v0.5.0, ggforce v0.4.2.<br>Code generated for data processing and the production of figures have been deposited in Zenodo (https://zenodo.org/records/10836320).

For manuscripts utilizing custom algorithms or software that are central to the research but not yet described in published literature, software must be made available to editors and reviewers. We strongly encourage code deposition in a community repository (e.g. GitHub). See the Nature Portfolio guidelines for submitting code & software for further information.

Data

Policy information about availability of data

All manuscripts must include a data availability statement. This statement should provide the following information, where applicable:

- Accession codes, unique identifiers, or web links for publicly available datasets
- A description of any restrictions on data availability
- For clinical datasets or third party data, please ensure that the statement adheres to our policy

The following databases were used in this study: GTDB v2.2.1, NCBI, SRA, KEGG, RAST, Silva v138.1, Swiss-Prot v2023.05, KEGG v2022.07.6, ROCker v1 (https://rast.nmpdr.org). The sequencing data generated in this study have been deposited in the NCBI database under accession number PRJNA951658. The 16S rRNA amplicon sequencing data generated from 6th and 9th generation transfer cultures are deposited under SRA accessions SRR24215177 and SRR24083098. The metagenome raw data generated from co-culture EV and Serratia sp. MF have been deposited under SRA accessions SRR24709127 and SRR24709126. The El Verde soil metagenome raw data were generated in a prior study36 and deposited in the Qiita database under study 15067. The 16S rRNA gene sequences of Desulfosporosinus nitrosoreducens strain PR and Serratia sp. MF have been deposited under Genebank accessions OR076434 and OR076433. The draft genomes of Desulfosporosinus nitrosoreducens strain PR and Serratia sp. MF are available under GenBank accession numbers GCA_030954495.1 and GCA_030954505.1. The metabolomics raw data have been deposited in the MassIVE database under accession number MSV000094351. Source data are provided with this paper.

Research involving human participants, their data, or biological material

Policy information about studies with human participants or human data. See also policy information about sex, gender (identity/presentation), and sexual orientation and race, ethnicity and racism.

Reporting on sex and gender	N/A
Reporting on race, ethnicity, or other socially relevant groupings	N/A
Population characteristics	N/A
Recruitment	N/A
Ethics oversight	N/A

Note that full information on the approval of the study protocol must also be provided in the manuscript.

Field-specific reporting

Please select the one below that is the best fit for your research. If you are not sure, read the appropriate sections before making your selection.

☐ Life sciences ☐ Behavioural & social sciences ☒ Ecological, evolutionary & environmental sciences

For a reference copy of the document with all sections, see nature.com/documents/nr-reporting-summary-flat.pdf

Ecological, evolutionary & environmental sciences study design

All studies must disclose on these points even when the disclosure is negative.

Study description	Our study started from a N2O-reducing microcosm that had been maintained over 16 month. The overall experimental design is explorative, aiming to search for microorganisms capable of low pH N2O reduction. From the microcosm, we applied iterative strategy to explore feasible medium composition to obtain solids-free transfer culture. Upon a co-culture became available, we integrate metagenomic and metabolomic approach into physiology characterization. We firstly design a single factor (N2O supply) experiment to demonstrate the N2O-dependent growth of Desulfosporosinus nitrosoreducens. Consequently, we compare the extracellular metabolome of axenic Serratia and co-culture to reveal an interspecies nutritional interaction. Our findings provide unambiguous evidence that low pH N2O reducers exist in acidic soils, and reconcile the knowledge gap between envisaged and actual N2O reduction potential in acidic soils.
Research sample	The tropical forest soils have a characteristic pH of acidic soils (pH <5), and harbor a diverse nosZ-carrying microorganisms. Thus, the tropical forest soils serve as an representative inoculum for enrichment culturing acidophilic N2O reducing microorganisms. Enrichment culturing was applied to obtain a co-culture, from the acidic tropical soils, capable of N2O reduction at pH 4.5.
Sampling strategy	At least three biological replicates were collected for phenotype, physiological and quantitative PCR analyses. Based on the growth performance and excellent reproducibility, representative samples were collected for 16S rRNA gene Sanger sequencing,amplicon sequencing, metagenomic sequencing and metabolomic analyses.
Data collection	Establishment of co-culture, physiology and molecular characterization and bioinformatic analyses were conducted by Guang He. Metabolomic analyses were performed by Katarina A. Jones at Biological and Small Molecular Mass Spectrometry Core (BSMMSC).
Timing and spatial scale	Establishment of the co-culture material took two-year (from 2020 Jan. to 2022 Jan.). Preliminary growth experiments determined

the incubation period for the experiments presented in our manuscript. When grown with pyruvate, H2 and N2O, co-culture EV batch incubations were maintained for one month; when grown with amino acid mixture, H2 and N2O, co-culture EV cultures were maintained for one week. Thus, the growth experiments of co-culture typically took from one week to one month, depending on the substrates provided. Extended incubation periods of up to two-month were accounted for negative observations.

Data exclusions

No data was excluded for analyses.

Reproducibility

All experiments were independently repeated at least once to ensure reproducibility.

Randomization

N/A, this study is explorative, and generates hypothesis based on experimental observation, and test hypothesis as they arise.

Blinding

N/A, we determined that co-culture EV is stable, and the community would not change over transfer procedure. We have done preliminary characterization of the phenotype and we have expectation on the co-culture performance under different substrate combinations.

Did the study involve field work? ☐ Yes ☒ No

Reporting for specific materials, systems and methods

We require information from authors about some types of materials, experimental systems and methods used in many studies. Here, indicate whether each material, system or method listed is relevant to your study. If you are not sure if a list item applies to your research, read the appropriate section before selecting a response.

Materials & experimental systems

n/a	Involved in the study
☒	☐ Antibodies
☒	☐ Eukaryotic cell lines
☒	☐ Palaeontology and archaeology
☒	☐ Animals and other organisms
☒	☐ Clinical data
☒	☐ Dual use research of concern
☒	☐ Plants

Methods

n/a	Involved in the study
☒	☐ ChIP-seq
☒	☐ Flow cytometry
☒	☐ MRI-based neuroimaging

Plants

Seed stocks

N/A

Novel plant genotypes

N/A

Authentication

N/A
